# Platelet Activation: The Mechanisms and Potential Biomarkers

**DOI:** 10.1155/2016/9060143

**Published:** 2016-06-15

**Authors:** Seong-Hoon Yun, Eun-Hye Sim, Ri-Young Goh, Joo-In Park, Jin-Yeong Han

**Affiliations:** ^1^Department of Biochemistry, Dong-A University College of Medicine, 26 Daesingongwon-ro, Seo-gu, Busan 49201, Republic of Korea; ^2^Department of Laboratory Medicine, Dong-A University College of Medicine, 26 Daesingongwon-ro, Seo-gu, Busan 49201, Republic of Korea

## Abstract

Beyond hemostasis and thrombosis, an increasing number of studies indicate that platelets play an integral role in intercellular communication, mediating inflammatory and immunomodulatory activities. Our knowledge about how platelets modulate inflammatory and immunity has greatly improved in recent years. In this review, we discuss recent advances in the pathways of platelet activation and potential application of platelet activation biomarkers to diagnosis and prediction of disease states.

## 1. Introduction

Platelets play key roles in hemostasis. Platelets are generated from the nucleated precursor cells known as megakaryocytes in the bone marrow and enter the bloodstream without nuclei. Megakaryopoiesis, the complex process that megakaryocytes mature, differentiate, and generate polypoid megakaryocytes, is unique to mammalian cells. Megakaryopoiesis and thrombopoiesis are controlled by multiple cytokines and growth factors, although thrombopoietin is the key regulator. Mature megakaryocytes restructure their cytoplasm and extend pseudopodial projections referred to proplatelets, through cells of the sinusoidal endothelial layer and shed platelets into the circulation. A steady supply is secured by a continuous production and clearance of 10^11^ platelets daily to maintain 150–400 × 10^9^/L of blood level [1–3].

As small anucleate cellular fragments, platelets are metabolically active cells, containing numerous functional organelles such as endoplasmic reticulum, Golgi apparatus, and mitochondria. They have a wide array of surface receptors and adhesion molecules and contain numerous granules. Since they have mRNA, platelets can synthesize a limited amount of proteins. However, there are a vast number of molecules preformed and inherited from the megakaryocytes in platelets, which can be released upon activation [2–5].

The basic function of platelets is rapidly binding to damaged blood vessels, aggregates to form thrombi, and prevents excessive bleeding. However, activated platelets also aggregate at the site of atherosclerotic plaque rupture or endothelial cell erosion, stimulating thrombus formation and promoting atherothrombotic disease [5, 6]. Recent studies indicated that antiplatelet medications affect host immunity and modify platelet response to inflammation, reducing mortality from infections and sepsis [7]. Therefore, there is an increasing evidence that platelets have a central role in the host inflammation and immune responses [1, 2, 4–10]. In this review, we discuss recent advances in the knowledge of platelet activation and potential application of platelet activation biomarkers to diagnosis and prediction of disease states.

## 2. Platelet Activation Pathways

Platelet activity is primarily associated with the initiation of coagulation cascades. Platelet adhesion to the extracellular matrix is the first step in primary hemostasis. Under the conditions of high shear, von Willebrand factor (vWF) forms a bridge between exposed collagen and the platelet glycoprotein (GP) Ib-IX-V receptor complex on the platelet membrane [5, 6, 11]. Exposed collagen also binds directly to platelet GP Ia/IIa and GP VI receptors. Activated during this process, platelets change shape and release the contents of their granules. Active GP IIb/IIIa receptor has central role in mediating platelet aggregation. Bound fibrinogen or vWF to GP IIb/IIIa cross-links platelets and contributes to thrombus stabilization [5, 6, 11, 12].

Platelet activation is stimulated by bound platelet secretion products and local prothrombotic factors such as tissue factor. Multiple pathways can lead to platelet activation. There are two principle activating pathways in platelets [5, 6, 9, 11–14]. GP Ib-IX-V, GP VI, or C-type lectin-like receptor 2 (CLEC-2) are all membrane glycoproteins exclusively expressed in platelets and megakaryocytes and have closely related signal transduction pathways. GP VI is thought to be the major signaling receptor involved in platelet activation on exposed collagen. Following GP VI interactions with collagen, platelets initiate strong activation and release the content of *α*- and dense granules. Recently, CLEC-2 has been identified as mediating the potent platelet activation response to rhodocytin, a platelet-activating snake venom [13–15]. Platelet activation by GP VI and CLECL-2 is through receptors containing the immunoreceptor tyrosine-based activation motif (ITAM) sequence. There is a growing evidence that platelets also function independently of full aggregation to regulate vascular permeability and development. Many of these novel platelet function depend on ITAM signaling rather than via activation through G protein-coupled receptors (GPCR).

Most soluble agonists released by activated cells such as ADP, thromboxane A_2_ (TxA_2_), and thrombin trigger platelet activation through GPCR [2, 5, 9–13]. This increases the cytosolic calcium concentration and activates specific signaling pathways. ADP released from damaged endothelial cells and activated platelets acts on platelet P2Y_1_ and P2Y_12_ GPCR, which causes further platelet activation and release of ADP. P2Y_12_ receptor sustains platelet activation in response to ADP and therefore has a central role in this process. TxA_2_ produced and released by stimulated platelets also activates further platelets via GPCR, thereby promoting plug formation.

Thrombin is the most strong platelet agonist and also responsible for converting fibrinogen into fibrin to stabilize the platelet plugs [5, 6, 9, 13]. Thrombin activates platelets through protease-activated receptors (PAR) on the platelet surface via GPCR. PAR1 mediates human platelet activation at low thrombin concentration, while PAR4 requires higher concentration of thrombin for platelet activation. Signaling via PAR4 is available for a protective mechanism in situations such as trauma to contribute to arrest bleeding. Other agonists like epinephrine, prostaglandin E_2_, and serotonin can also utilize GPCR to potentiate platelet responses [13].

All these platelet signaling events converge upon the final common pathway of platelet activation, the functional upregulation of integrin adhesion receptors [5, 11–13]. The most important is the activation of the GP IIb/IIIa receptor which results in the cross-linking of fibrinogen or vWF between receptors, leading to platelet aggregation. This promotes further the recruitment of additional platelets to the site of vascular injury, allowing the subsequent thrombus formation.

## 3. Cellular Interactions

Activated platelets secret a number of inflammatory mediators that have no apparent role in hemostasis. Under hemostatic conditions, platelets generally do not bind to leukocytes. However, when activated, platelets adhere to neutrophils and monocytes, and interactions with lymphocytes have also been identified [2, 5, 6, 8–12]. Platelets interact with the vascular endothelium and leukocytes and link inflammation, thrombosis, and atherogenesis. Recently platelet serotonin in dense granules has been shown to play an important role in neutrophil rolling and adhesion to the endothelium [5].

Binding between platelets and other cell types is primarily mediated by P-selectin (also known as CD62p). Upon activation, platelets express large amount of P-selectin which is rapidly mobilized from *α*-granules to the platelet surface [8–12]. P-selectin via its ligand, P-selectin glycoprotein ligand-1 (PSGL-1), has a central role in the interactions between platelets, leukocytes, and endothelial cells. Monocytes, neutrophils, eosinophils, and hematopoietic progenitor cells have all been reported to express PSGL-1 [5, 6, 10]. P-selectin cross-links platelets and leukocytes and is a major mediator of platelet-leukocyte aggregate formation, thereby upregulating release of proinflammatory cytokines and adhesion to endothelium.

Activated platelets also express CD40L (also known as CD154). Platelets expression of CD40L has been shown to affect dendritic cells as well as B and T lymphocytes, suggesting that it provides a communicative link between innate and adaptive immunity [5, 6, 8–12]. It also interacts with CD40 on endothelial cells to promote secretion of chemokines and expression of adhesion molecules. Furthermore, platelets are known as the predominant source of soluble CD40L (sCD40L), which can induce vascular cells to express E-selectin and P-selectin and release interleukin- (IL-) 6 [5].

Platelet factor 4 (PF4) or also known as CXC chemokine ligand 4 (CXCL4) is one of the most abundant proteins contained in platelet *α*-granules. In addition to a role in thrombosis and hemostasis, PF4 has a broad range of activities related to innate immunity [5, 6, 9]. PF4 promotes neutrophil granule release and adhesion to endothelial cells, mediated by L-selectin and leukocyte function-associated antigen-1 (LFA-1). PF4 also prevents monocyte apoptosis, promotes monocytic differentiation into macrophages, and induces phagocytosis and generation of reactive oxygen species (ROS) [5]. PF4 and RANTES (regulated on activation, normal T cell expressed and secreted) form heterodimers, leading to promote monocyte recruitment to the endothelium. RANTES also known as chemokine ligand 5 (CCL5) is a chemokine that has a role in atherosclerosis and found in large quantities in platelet *α*-granules [5, 6].

Platelets also participate in pathogen capture and sequestration. When platelets bind to neutrophils, they trigger the release of chemokines and the formation of neutrophil extracellular traps (NET) [5, 8–10, 12]. NET are released from activated neutrophils and comprise an extrusion of DNA, DNA-associated nuclear proteins, such as histones and serine proteases including neutrophil elastase. Pathogen related factors such as lipopolysaccharide (LPS) stimulate both neutrophils and platelets, leading to NET release and activation of neutrophil *α*M*β*2 integrin (Mac-1 that binds to platelet GP Ib*α*) and activating platelets to express P-selectin (that binds to neutrophil PSGL-1). The association between platelets and leukocytes bringing on the release of intravascular NET provides the new opportunities for the development of diagnostic assays and prognostic indicators in inflammatory or septic conditions [5, 8–10, 12].

## 4. Platelet Activation Markers

Platelets activation markers can be well studied by enzyme-linked immunosorbent assay (ELISA) or Western blot. But flow cytometer is currently the best standardized method to study platelet function. Although flow cytometry requires sophisticated equipment and available monoclonal antibodies, it has several advantages including small volume of whole blood and independence of platelet count [2, 3, 11]. Flow cytometry allows the analysis of the expression of platelet activation markers and receptors on individual platelets as well as the quantitation of associates between platelets and other blood cells [16–20].

### 4.1. Platelet Granules

Platelets contain at least three different types of granules: *α*-granules, dense granules, and lysosomes [4, 5, 9, 10]. Granule-stored mediators include coagulation and angiogenic factors, adhesion molecules, cytokines, and chemokines. Although preformed mediators allow for rapid release following platelet activation, platelets also possess the ability to synthesize additional mediators. Many functions of platelets result from the vast array of preformed cell membrane and soluble mediators contained within granules.

*α*-Granules are the most abundant granules, and there are about 50–80 *α*-granules per platelet. The contents are known to include adhesive glycoproteins such as P-selectin, fibrinogen, and vWF, coagulation factors, mitogenic factors, angiogenetic factors, fibrinolytic inhibitors, immunoglobulins, granule membrane-specific proteins such as P-selectin and CD63, and various chemokines including PF4 (CXCL4) and RANTES [5, 9, 10]. Many *α*-granule-derived mediators have an important role in hemostasis, but they also have a significant role in innate immunity, either by modulating the expression platelet adhesion receptors interacting with leukocytes or by releasing cytokines that affect leukocyte function [5, 9, 10].

Dense granules store a variety of hemostatically active nonprotein molecules which are secreted during platelet activation. Those include catecholamines such serotonin and histamine, ADP, ATP, and calcium [2, 5, 10]. The third type of granule, platelets lysosomes, contain glycosidases, acid proteases, and cationic proteins that have a bactericidal activity [5, 10].

### 4.2. Platelet Cytokines and Adhesion Molecules

Activated platelets release various chemokines such as CXCL1, PF4 (CXCL4), CXCL5, CXCL7, IL-8 (also known as CXCL8), CXCL12, macrophage inflammatory protein- (MIP-) 1*α* (also known as CCL3), and RANTES (CCL5) [5, 6, 9, 10, 21]. The major effect of these cytokines is to regulate leukocyte movement, migration from the vasculature into the tissues, and other proinflammatory functions like phagocytosis and generation of ROS [5, 21]. The proinflammatory cytokine IL-1*β* released by activated platelets has also been suggested to have a major role in vascular inflammation.

Platelets express numerous adhesion molecules and ligands that facilitate interactions between platelets, leukocytes, and endothelium [5, 6, 9, 10, 22]. Platelets express large amount of P-selectin, which has a key role in linking hemostasis and inflammation [5, 10]. Integrins comprise a large family of heterodimeric transmembrane receptors that are formed by noncovalent association of different *α* and *β* chains. They mediate interactions with extracellular matrix (ECM) molecules and adhesion molecules on other cells [10, 22]. Platelets express a number of *β*1 and *β*3 integrins, including *α*2*β*1 known as very late antigen-2 (VLA-2, collagen receptor), *α*5*β*1 (VLA-5, fibronectin receptor), *α*6*β*1 (VLA-6, laminin receptor), *α*IIb*β*3 (fibrinogen receptor), and *α*V*β*3 (vitronectin receptor). This complex array of adhesion molecules and ligands allow and facilitate platelets to bind to a number of diverse cellular and structural targets under shear conditions [10].

### 4.3. Platelet-Leukocyte Aggregates

The interaction between platelets, leukocytes, and endothelium can occur in various ways. Activated platelets bind to leukocytes through P-selectin, GP IIb/IIIa, and CD40L [5, 6, 8–10, 12]. In addition to serving as a platform to which leukocytes can adhere, platelets also have the capacity to modulate the expression and activation of adhesion molecules on other cell types such as neutrophils, monocytes, lymphocytes, and endothelium. Platelet-neutrophil and platelet-monocyte aggregates have been detected in the blood of humans with a variety of diseases and are now considered as one of the most sensitive markers related to platelet activation [17–19]. They may reflect a prothrombotic state and are reported to be associated with acute coronary syndrome, systemic inflammatory conditions, and neoplastic and autoimmune diseases.

## 5. Clinical Applications

In recent years, platelets have emerged as important markers for various types of diseases. They are multifunctional blood particles and now regarded to be very important clinical targets for many disease pathophysiology. In addition to playing a central role in normal hemostasis and thrombosis, platelets can make important contributions to host inflammatory and immune responses to infection or injury. Under uncontrolled pathological conditions, they have profound roles in pathogenic processes underlying atherosclerosis and cardiovascular diseases, uncontrolled inflammation, tumor metastasis, and neurodegenerative diseases including Alzheimer's disease [17–25].

Upon activation, platelet surface P-selectin is overexpressed, and platelets secret their granule contents into circulation. Several markers of platelet activation such as P-selectin, CD40L, PF4, and GP IIb/IIIa have been identified to correlate with the presence of inflammation and atherosclerosis [17–19, 26]. Because these markers have relatively short detectability in the circulating blood, platelet-monocyte aggregates have emerged as markers for platelet activation [18, 19]. Platelet-monocyte aggregates have longer persistence in peripheral blood and were shown to be more sensitive markers of in vivo platelet activation than other platelet surface markers. They serve to link coagulation and the development of atherosclerosis.

Microparticles are plasma membrane-derived vesicles ranging in diameter from 0.1 to 1.0 *μ*m. Platelets and megakaryocytes are the primary source of microparticles in the blood circulation. They can carry nuclear and cytoplasmic components from their parent cells and transfer this information to affect nearby or distant cells [20, 25, 27]. Platelet microparticles contain proteins, lipids, and RNA derived from their precursor cells. Therefore, circulating platelet microparticles can be considered as biologic markers associated with platelet activation. Previous studies have reported that the levels of circulating platelet microparticles were increased in patients with various diseases such as hypertension, atherosclerosis, and stroke [27]. A few studies have demonstrated a predictive value of platelet microparticles in thrombotic diseases [20]. Although important efforts in standardizing the preanalytical and analytical variables have been developed, the provision of more accurate quantification technologies can help to advance the field.

NET consist of filamentous DNA (chromatin) arrayed with histone proteins and several antibacterial components extruded from activated neutrophils during inflammatory responses [5, 8–10, 12, 23, 24]. Once released into the circulation, NET is supposed to serve to trap, restrain, and neutralize invading microbes. Activated platelets can induce NET formation, form platelet-neutrophil and platelet-platelet aggregates, and be trapped by NET. This association between platelets and leukocytes leading to the release of intravascular NET provides new opportunities for the development of diagnostic assays in severe inflammatory diseases [10]. In conclusion, the collaborative involvement of platelets and neutrophils in the inflammation and cardiovascular diseases provide a novel link between inflammation and thrombosis [23, 24].

## 6. Conclusions

Platelets normally circulate through the vasculature in an inactive, nonadhesive state. In response to an injury to the endothelium, bacterial infection, or alteration to normal blood flow, platelets rapidly decelerate, roll on the injured endothelium, and firmly adhere. They play a central role in normal hemostasis by maintaining vascular patency. However, emerging evidence demonstrates that platelets are far more complex than previously regarded, equipped with elaborate intracellular machinery.

Now recognized as key players in inflammatory and innate immune responses, platelets have the capacity to interact with almost all immune cells. Understanding the platelet activation pathways and potential biomarkers could promise new diagnostic and therapeutic possibilities in monitoring the disease activities and responses to treatment.
